# Educational impact of a cost-efficient porcine model for toe amputation simulation training: Enhancing amputation education

**DOI:** 10.1016/j.jpra.2025.09.007

**Published:** 2025-09-14

**Authors:** Ameen Mahmood, Cheuk Ying Kyleen Kiew, Anuska Shah, Rananjay Singh, Marya Abuarqoub, Logan Barnes, Matthew Wordsworth

**Affiliations:** a School of Medicine, Imperial College London, London, United Kingdom; b School of Medicine, St George’s University of London, London, United Kingdom; c Department of Surgery, Imperial College NHS Trust, London, United Kingdom

**Keywords:** Amputation education, Porcine simulation model, Surgical skills training, Undergraduate medical education, Low-cost surgical training

## Abstract

**Introduction:**

Undergraduate amputation education is underrepresented, limiting exposure to training. Porcine simulation models, known for their anatomical similarity to humans and cost-efficiency, have gained popularity in surgical education. This study evaluates the educational impact of a low-cost porcine toe amputation simulation model in enhancing theoretical knowledge, technical skills, and anatomical understanding.

**Methods:**

A prospective cohort study was conducted through a structured workshop—including a didactic lecture on anatomy and surgical principles, followed by hands-on practice with porcine models. Pre-and post-workshop surveys assessed theoretical knowledge, surgical confidence, instrument familiarity, and post-operative management using Likert scales and knowledge-based assessments. Technical skills were evaluated using Objective Structured Assessment of Technical Skills (OSATS). Statistical analysis employed Wilcoxon Signed-rank and Mcnemar’s test.

**Results:**

A total of twenty seven participants (24 medical students and three resident doctors) with no prior toe amputation experience participated. Theoretical knowledge improved significantly from 1.52 ± 0.98 to 3.81 ± 1.04 (*p* < 0.01), and surgical confidence increased from 1.48 ± 0.98 to 3.44 ± 1.19 (*p* < 0.01). Familiarity with anatomical structures rose from 1.78 ± 0.97 to 3.22 ± 1.19 (*p* < 0.01). OSATS scoring showed significant improvements in all areas (*p* < 0.01). Participants rated workshop effectiveness at 4.19 ± 0.74 and recommendation likelihood at 4.52 ± 0.64.

**Conclusion:**

The simulation model significantly improves knowledge and technical skills. Future workshops could incorporate psychological and physiological care to provide a comprehensive learning experience.

## Introduction

Amputations represent a significant and growing global health challenge, rooted in both chronic disease and traumatic injury. Globally, an estimated 65 million people live with limb loss, and approximately 1.5 million new amputations occur annually, largely attributed to diabetes, peripheral vascular disease, road traffic accidents, occupational injuries, landmine explosions, and armed conflict.^1^^,^^2^ In low-and middle-income countries, trauma and conflict remain the predominant causes, often affecting young adults and children.^3^ In the United Kingdom (UK), around 5000 major limb amputations are performed annually, primarily due to chronic vascular pathology.^4^ Diabetes mellitus, which affects over 4.6 million people in the UK, increases the risk of amputation nearly nine-fold compared with non-diabetic individuals—often leading to distal amputations, particularly of the toes.^5^, ^6^, ^7^

Optimal management of amputations necessitates coordinated multidisciplinary care involving vascular and plastic surgeons, anesthetists, rehabilitation physicians, physiotherapists, occupational therapists, prosthetists, specialist nurses, and psychologists.^4^

Despite amputations being frequently performed and amputee patients being managed across multiple specialties, undergraduate exposure remains limited. A national survey of UK final-year medical students reported that only 29.1 % received formal teaching on amputations, and just 18 % had direct clinical exposure to amputee patients. The most frequently cited barrier (79.4 %) was the lack of dedicated teaching, with only 10.5 % of students reporting confidence in providing adequate amputation care upon graduation.^8^

Simulation-based learning has proven effective in addressing educational deficiencies in medical training.^9^ By replicating clinical scenarios in controlled environments, simulators allow learners to practice surgical skills, refine decision-making, and build confidence without the risks associated with patient care. Porcine models, in particular, have emerged as highly effective tools in surgical education due to their anatomical similarities to humans and their cost-efficiency.^10^ They have been successfully used in flap reconstruction and trauma management.^11^, ^12^, ^13^ Current literature reflects a notable lack of dedicated amputation simulation models, highlighting an important gap in available training resources.^14^

To address this unmet educational need, we developed a porcine toe amputation model. The model closely mimics human digit anatomy, providing opportunities to practise essential surgical steps such as incision planning, tissue dissection, and joint exposure. As well as reinforcing intraoperative decision-making, surgical judgement, and perioperative care principles.

This study evaluates the educational impact of the porcine toe amputation model, through a structured workshop, on the knowledge, skills, and confidence of medical students and resident doctors. Through a combination of didactic and simulation training, the workshop aimed to address the current gap that exists within amputation education.

## Methods

### Study design

This was a prospective cohort study, utilizing pre-and post-intervention assessments to evaluate changes in knowledge, confidence and technical skills. The study design was informed by previous models of simulation-based surgical education within plastic and vascular surgery.^14^^,^^15^ This study was reported in accordance with the STROBE (Strengthening the Reporting of Observational Studies in Epidemiology) guidelines.

### Setting and ethical approval

This study was conducted in a controlled simulation environment at Imperial College London, UK in November 2024. The workshop was delivered as a single 120-min session through Imperial College Plastic Reconstructive and Aesthetic Surgery Society (iPRAS). The study received ethical approval from Imperial College Education Ethics Review Process (EERP2425–065).

### Population and recruitment process

The workshop was open to all UK medical students and healthcare professionals, promoted in coordination with iPRAS. Participants were recruited through societies, mailing lists and social media. Eligible individuals included those with prior experience in basic suturing but no experience in toe amputation surgery, self-reported via a pre-workshop survey. Observational exposure alone did not meet exclusion criteria.

### Simulation model

**Figure 1:**
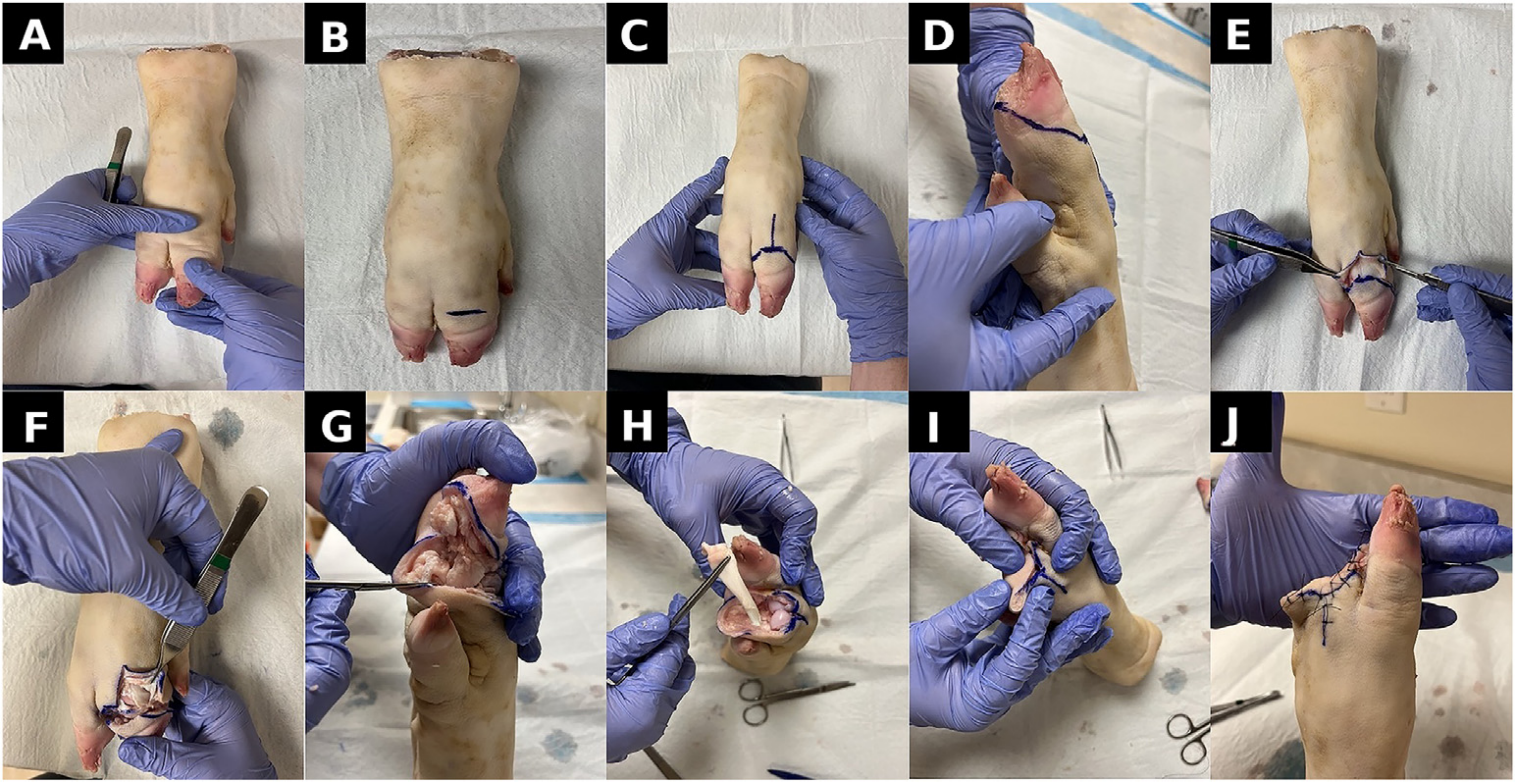
Step-by-step porcine toe amputation simulation procedure. (A) Anatomical joint line identified via plantarflexion and dorsiflexion. (B) Initial marking of the joint using an indelible surgical pen. (C) Racket-shaped incision drawn, incorporating a long plantar-based flap. (D) Medial view showing extension of the incision and anatomical landmarks. (E) Circumferential incision initiated using a scalpel. (F) Dissection to expose the joint and collateral ligaments. (G) Plantar view showing full exposure of the metatarsophalangeal joint and phalangeal surfaces. (H) Division of the long flexor tendon as proximally as possible. (I) Planning of the soft tissue closure with early approximation of plantar and dorsal flaps. (J) Completed closure using simple interrupted sutures with good tissue alignment.

The simulation model utilized porcine trotters to closely replicate the anatomical characteristics of human toes.^16^ A 10-step procedural protocol was followed, covering joint identification, racket incision planning, dissection, tendon division, and flap closure. Detailed procedural steps are summarized in Table 1, with a visual step-by-step demonstration presented in Figure 1.

**Table 1:** Porcine toe amputation simulation: step-by-step guide. A detailed breakdown of the 10 procedural steps performed using the porcine model, from joint identification and incision to dissection and layered closure. This protocol was followed by all participants during the simulation.

**Step**	**Description**
1	The porcine trotter is positioned with the plantar surface facing down. The metatarsophalangeal joint is identified by plantarflexing and dorsiflexing the digit.
2	The joint line is marked using an indelible surgical pen to guide incision placement.
3	A racket-shaped incision is drawn, ensuring a long plantar-based flap to allow tension-free closure.
4	A circumferential incision is performed with a scalpel through the skin and soft tissue to the level of the joint capsule.
5	The joint capsule is exposed and incised carefully to reveal underlying bony structures.
6	Collateral ligaments on both medial and lateral aspects are divided to enable joint disarticulation.
7	The long flexor tendon is optionally identified and transected proximally to simulate tendon management.
8	Additional skin may be excised as needed to optimize tissue closure.
9	The wound is approximated with simple interrupted sutures, with care to secure the plantar flap.
10	Closure is completed, assessing for flap alignment, minimal gapping, and overall wound integrity. Dog-ear revisions may be performed if necessary.

### Training protocol

The workshop followed a structured three-stage format:

1. Interactive theoretical session: A 45-min didactic lecture by a consultant plastic surgeon who specializes in amputations. This session provided participants with foundational knowledge, covering surgical anatomy relevant to toe amputation, as well as the clinical indications for amputation and the underlying reasoning behind each procedural step. The lecture also addressed post-operative care, including wound healing, stump management, infection control, and rehabilitation.
2. Demonstration: A step-by-step live demonstration of toe amputation on the porcine model. The demonstration included anatomical surface marking, dissection techniques, identification of key structures, and closure principles.
3. Simulation practice: Participants then undertook hands-on practice using porcine trotters to simulate toe amputation. Each delegate performed the procedure using standard surgical instruments. Real-time supervision was provided by surgical trainees and consultants with experience in amputations.

### Assessment protocols

A mixed-methods framework assessed the model’s educational impact. Participants completed pre- (Appendix A), and post-workshop surveys (Appendix B) via the online platform Qualtrics. These surveys were designed to capture both quantitative and qualitative data across multiple domains, including theoretical knowledge, technical competence, and perception of the simulation model. Impact was measured by comparing pre-and post-workshop scores to identify gains in knowledge, confidence, and procedural accuracy.

The survey instruments utilized a modified version of the Student Evaluation of Educational Quality (SEEQ) survey to assess satisfaction, model realism, educational effectiveness, and the likelihood of recommending.^17^ In addition procedural confidence and perception questions specific to the model utilized a confidence scale adapted from a five-point Likert tool used for assessing self-efficacy in surgical training.^18^

Integrated knowledge based assessments including multiple-choice questionnaires (MCQ) were objectively used to assess procedural understanding, identification of anatomical landmarks and surgical principles.

Post-training technical performance was evaluated using the OSATS, a validated tool for surgical skill assessment.^19^ OSATS included both global and task-specific criteria, scored on a one to five scale across domains such as instrument handling, economy of movement, and tissue management (Appendix C). Task-specific domains, developed with a consultant plastic surgeon who specializes in amputations, included surgical planning and markings, incision accuracy, dissection safety, joint exposure, suture and knot technique and the overall outcome (Appendix D). Assessors, comprising surgical trainees and consultants with prior experience in amputation surgery, were briefed in standardized scoring protocols to ensure consistency and minimize bias. Each assessor evaluated a subset of three participants using the same rubrics. As no participants had prior experience in toe amputations, pre-workshop OSATS scores were scored at a standardized baseline of one with a standard deviation (SD) = 0.

### Sample size and statistical analysis

A prior sample size calculation was performed using G*Power 3.1.^20^ Based on a Wilcoxon signed-rank test for matched pairs, a two-tailed alpha of 0.05, power of 80 %, and an anticipated moderate effect size (Cohen’s *d* = 0.6), the minimum required sample size was determined to be 24 participants.

Responses were handled in Microsoft Excel Version 16.91 and R Version 2025.05.0 + 496^21^ was used for statistical analysis. Descriptive statistics summarized demographic data and survey responses. Data analysis confirmed Cronbach's alpha of 0.8 for Likert-scale items, indicating good internal consistency. Normality was assessed using the Kolmogorov–Smirnov test. Paired *t*-test or Wilcoxon Signed-Rank Test was used to compare paired pre-and post-workshop scores depending on normality for Likert scale items. McNemar’s Test and Chi-squared was used for categorical improvements in knowledge-based assessments. Likert scale data was summarized using means and standard deviations (SD). Thematic analysis was used to evaluate qualitative feedback. Statistical significance was set at *p* < 0.05.

## Results

### Participant demographics

Pre-and post-workshop surveys were completed by 27 participants, including 24 medical students, one resident doctor, and two surgical trainees. Specifically, six were Year 1 students, 12 were in Year 2, and one from Year 3, one from Year 4, three participants were in their final year (Year 5), and one was intercalating. Among postgraduate attendees, one was a Senior House Officer and two were Core Surgical Trainees. All had prior suturing experience but no hands-on exposure to toe amputations.

### Theoretical knowledge and confidence

**Figure 2:**
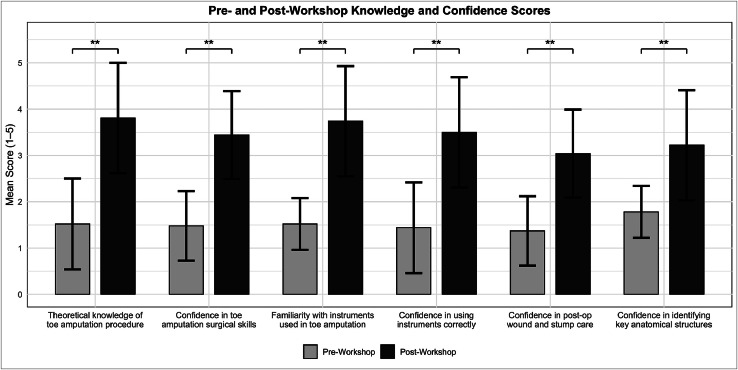
Pre-and post-workshop participant-rated scores on theoretical knowledge, procedural confidence, and instrument familiarity for toe amputation. Bars represent mean scores, with error bars indicating standard deviation. Statistically significant improvements were observed across all domains (Wilcoxon signed-rank test, *p* < 0.01), denoted by **. Maximum possible score = 5.

Following the workshop, participants demonstrated statistically significant improvements in self-reported confidence and knowledge across all measured domains (Figure 2). The mean score for perceived theoretical knowledge of toe amputation increased from 1.52 ± 0.98 to 3.81 ± 1.04 (*p* < 0.01). Confidence in identifying key anatomical structures such as joints, tendons, and bones improved from 1.78 ± 0.97 to 3.22 ± 1.19 (*p* < 0.01). Familiarity with the required instruments rose from 1.52 ± 0.75 to 3.74 ± 0.90 (*p* < 0.01). Participants also expressed increased confidence in managing post-operative care, including wound care, stump care, and dressing, with scores improving from 1.37 ± 0.56 to 3.04 ± 1.29 (*p* < 0.01).

**Figure 3:**
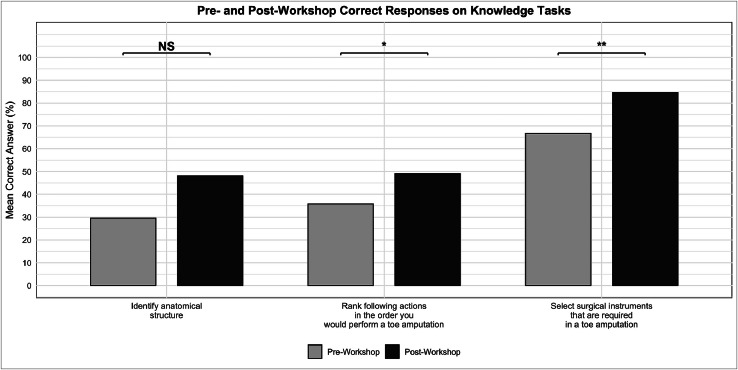
Pre-and post-workshop participant-rated correct responses for knowledge-based questions on toe amputation. Bars represent the mean correct percentage for each question. Statistically significant improvements are marked with asterisks (*p < 0.05, **p < 0.01); NS indicates non-significant differences.

Objective knowledge-based assessments showed increases across procedural ordering, anatomical structure identification, and instrument selection tasks (Figure 3). Participants were asked to correctly rank nine procedural steps involved in a toe amputation, the mean number of accurately ordered steps improved from 35.8 % (3.22 ± 1.78) to 49.0 % (4.41 ± 2.59) post-workshop (*p* = 0.02). Correct identification of an anatomical structure rose from 29.6 % to 48.1 % (*p* = 0.07). When asked to select appropriate surgical instruments used in toe amputation, out of 12, scores improved from 66.7 % (8.00 ± 2.73) to 84.6 % (10.15 ± 2.14) (*p* < 0.01).

### Technical skills improvement

Participants showed significant improvements in surgical and instrument-handling confidence. Confidence in performing a toe amputation increased from 1.48 ± 0.98 to 3.44 ± 1.19 (*p* < 0.01). Confidence in the correct use of instruments improved from 1.44 ± 0.70 to 3.50 ± 0.95 (*p* < 0.01).

**Figure 4:**
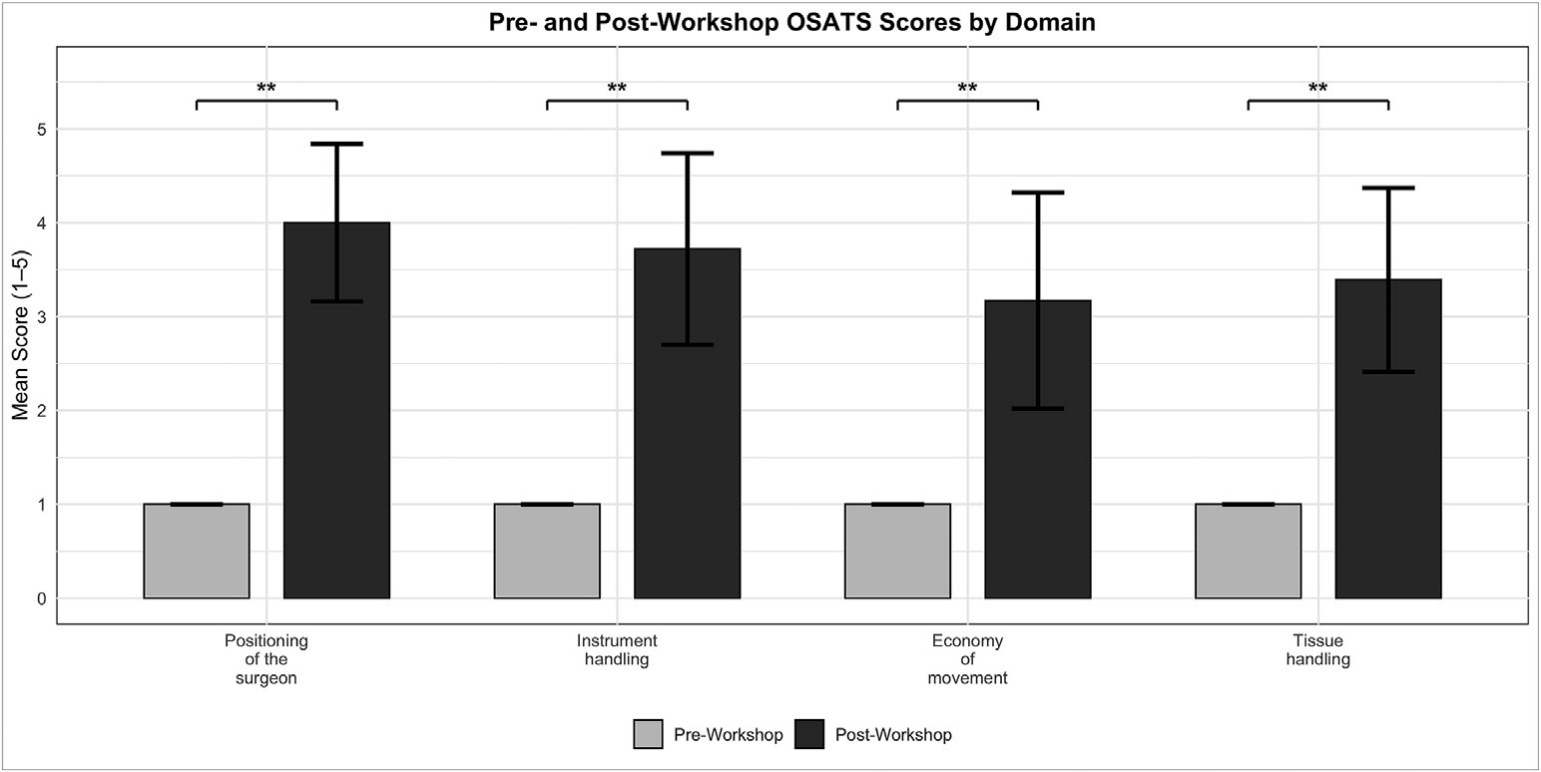
Pre-and post-workshop osats scores by domain. Mean OSATS scores for four technical domains before and after participation in a porcine toe amputation simulation workshop. Bars represent mean scores, with error bars indicating standard deviation. Statistically significant improvements were observed across all domains (Wilcoxon signed-rank test, *p* < 0.01), denoted by **. Maximum possible score = 5.

Objective technical performance was assessed using OSATS, with significant improvements observed across all global and task-specific domains. Post-workshop global assessment scores improved significantly across all domains, including surgeon positioning (4.00 ± 0.84), instrument handling (3.72 ± 1.02), economy of movement (3.17 ± 1.15), and tissue handling (3.39 ± 0.98), with all improvements being statistically significant (*p* < 0.01) (Figure 4).

**Figure 5:**
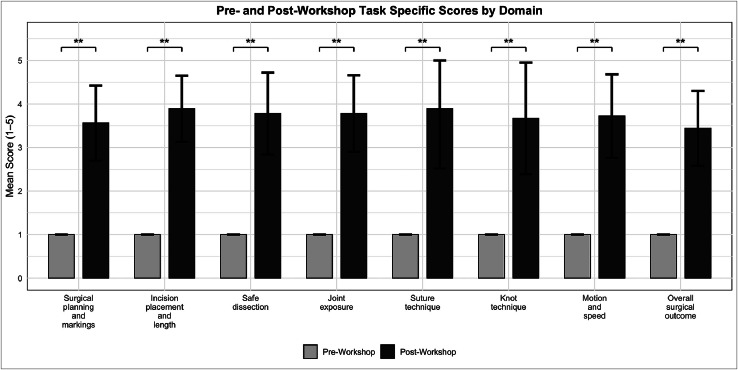
Pre-and post-workshop task specific scores by domain. Mean Task Specific scores for eight technical domains before and after participation in a porcine toe amputation simulation workshop. Bars represent mean scores, with error bars indicating standard deviation. Statistically significant improvements were observed across all domains (Wilcoxon signed-rank test, *p* < 0.01), denoted by **. Maximum possible score = 5.

Task-specific criteria also demonstrated statistically significant improvements. Post-workshop mean scores were 3.56 ± 0.86 for surgical planning and markings, 3.89 ± 0.76 for incision placement and length, 3.78 ± 0.94 for safe dissection, and 3.78 ± 0.88 for joint exposure. Technical suturing domains scored 3.89 ± 1.37 for suture technique and 3.67 ± 1.28 for knot technique. Motion and speed was rated at 3.72 ± 0.96, and the overall surgical outcome—defined by flap alignment, minimal gapping, and edge smoothness—was scored at 3.44 ± 0.86. All improvements were statistically significant (*p* < 0.01) (Figure 5).

### Perceptions of the simulation model

#### Model fidelity

The realism of the porcine model in replicating human anatomy received a mean rating of 3.22 ± 0.89. Participants also evaluated the model’s utility for specific surgical tasks. Scores included 3.70 ± 0.78 for learning joint incision techniques, 3.81 ± 0.56 for surgical planning, 3.22 ± 0.93 for understanding skin and tissue anatomy, and 4.07 ± 0.73 for learning the overall procedural steps related to toe amputation.

#### Educational utility

**Figure 6:**
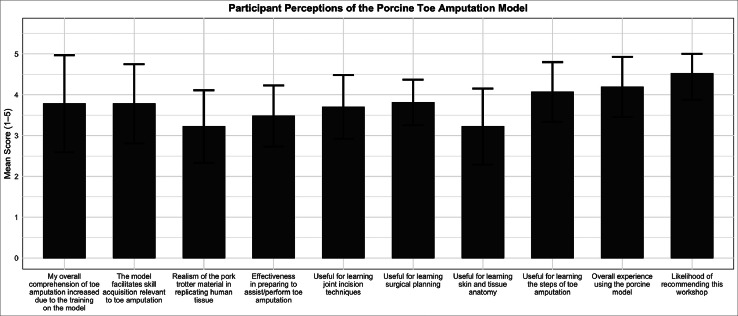
Participant-rated perceptions of the porcine toe amputation model following the workshop. Bars represent mean scores, with error bars indicating standard deviation across 10 domains, including realism, educational value, and procedural confidence. All items were rated on a five-point Likert scale.

Perceived overall comprehension of toe amputation increasing as a result of training on the model was rated 3.78 ± 1.19, and the model’s role in supporting skill acquisition was rated at 3.78 ± 0.97. The effectiveness of the workshop in preparing participants to assist or perform toe amputation in a clinical setting was rated 3.48 ± 0.75. The overall simulation experience was rated 4.19 ± 0.74, and the likelihood of recommending the model to others was high, with a mean recommendation score of 4.52 ± 0.64 (Figure 6).

#### Qualitative feedback

Thematic analysis of open-ended responses revealed three key themes:

Firstly, participants found the hands-on practice to be a valuable opportunity for experiential learning. Many appreciated the structured teaching and guidance provided by supervising clinicians. Secondly, a small number of participants reported difficulty in locating joint landmarks and requested more detailed anatomical instruction. Lastly, the workshop was overwhelmingly described as an enjoyable and educational experience. Representative comments included: “The hands-on practice was invaluable and well-supported by the teaching staff” and “This was a highly enjoyable learning experience with clear guidance.”

## Discussion

The findings of this study illustrate the educational value of simulation-based training particularly in the context of amputations. Participants demonstrated statistically significant improvements across all measured domains, including theoretical knowledge (1.52–3.81), procedural confidence (1.48–3.44), and technical ability (1.44–3.50), all *p* < 0.01. These results support the critical role of hands-on, experiential learning in fostering surgical skills and understanding and reflect current shifts toward active, skills-based education.^19^^,^^22^, ^23^, ^24^, ^25^, ^26^, ^27^

Notably, participants exhibited marked gains in procedural understanding, as reflected by improved accuracy in sequencing the steps of toe amputation (25 %–49 %, *p* = 0.02) and knowledge in the use of surgical instruments (66.7 %–84.6 %, *p* < 0.01). Although knowledge of procedural step order improved significantly (*p* = 0.02), accuracy remained relatively modest at 49 %. This likely reflects the high proportion of pre-clinical students in the cohort, many of whom would have had limited or no prior surgical exposure. This suggests that while simulation can enhance confidence and basic technical skills, repeated exposure and integration with ongoing surgical teaching may be required to consolidate procedural sequencing. OSATS scores improved significantly following the workshop, with statistical significance observed across both global performance metrics and task-specific domains (*p* < 0.01 for all criteria).

Importantly, the educational impact extended beyond technical proficiency. Participants reported significantly greater confidence in post-operative wound care and stump management (1.37–3.04, *p* < 0.01)—competencies critical to the holistic care of amputee patients. By equipping learners with practical, transferable skills that span the perioperative pathway, the workshop contributes to a more comprehensive and patient-centered approach.

### Curricular relevance and gaps in the literature

Although amputations are explicitly included in the Royal College of Surgeons of England (RCSEng) National Undergraduate Curriculum in Surgery—covering indications, operative techniques, complications, and rehabilitation^28^—these objectives are not consistently reflected in UK medical school training. A national survey of final-year UK medical students (*n* = 654) found widespread deficiencies in both formal teaching and clinical exposure. Simulation-based training reported by just 1.7 % of students. 74.8 % had never observed an amputation procedure and 82 % had not engaged in any hands-on procedural experience. Confidence in the psychological (43 %) and physiological (51 %) effects of amputations were low, and 71.9 % of respondents lacked familiarity with rehabilitation pathways or support services.^8^

These deficits are further illustrated by a recent systematic review of simulation training, which failed to identify any validated amputation models within the published literature.^14^ This absence of simulators represents a significant shortfall, particularly in light of the increasing emphasis on simulation-based education as a means of standardizing early skill acquisition. The urgency of addressing this gap is further highlighted by the increasing likelihood that resident doctors will encounter amputee patients during their foundation training, especially within vascular, diabetic, and trauma-related clinical settings.

The model offers a low-cost, easily accessible alternative with realistic tactile feedback, visual accuracy, and stepwise exposure to surgical planning, dissection, joint exposure, and wound closure. By embedding the model within a structured teaching programme, this initiative represents a scalable and curriculum-relevant approach to improving undergraduate competence in amputations.

### Global implications

Workshops of this nature are important in preparing current and future healthcare professionals to manage the complex and multidisciplinary demands of amputation care, with measurable improvements in both educational outcomes and technical skills. The need for such training is underscored by the rising global burden of limb loss—driven not only by increasing rates of diabetes and peripheral vascular disease but also by escalating numbers of traumatic amputations in conflict zones. Since 2022, it is estimated that over 50,000 military personnel in Ukraine have undergone amputations, while Gaza now has the highest number of child amputees per capita in the world.^29^^,^^30^

Our model offers a scalable and cost-effective training tool that is particularly well-suited for use in low-resource or humanitarian settings, where access to formal simulation centers may be limited, ultimately contributing to more equitable and consistent standards of amputation care delivery worldwide.

## Limitations and future directions

This study is subject to several limitations. It was conducted as a single-session workshop without longitudinal follow-up, limiting our ability to assess long-term knowledge retention or sustained technical competence. While immediate improvements in theoretical knowledge, procedural confidence, and OSATS scores were statistically significant, these gains may not equate to clinical readiness. It remains essential to evaluate whether such improvements persist over time and translate into real-world clinical performance. Future studies should explore the integration of follow-up assessments to evaluate skill decay, the need for refresher training and translation into clinical practice. Whether confidence development increases with repeated simulation exposure—or reaches a plateau—also remains an area for future investigation.^31^

Although simulation-based education offers a controlled and reproducible environment, it cannot fully mitigate variability in learner progression.^32^ Surgical skill acquisition is inherently individualized, with each trainee progressing along a unique learning curve. Participant heterogeneity may also have contributed to variability in outcomes, with younger students potentially requiring more guidance and achieving lower procedural accuracy. Between individuals, even of the same training level, prior knowledge or experience may have influenced both baseline knowledge and technical skills and as such the degree of improvement observed. Whilst this variability represents a limitation in terms of standardizing outcomes, it also reflects real-world conditions of cohorts, where prior surgical exposure and knowledge varies between individuals and training levels.^33^, ^34^, ^35^

The use of porcine introduces further limitations, the use of animal tissue may raise ethical concerns for some. Although the model provides a practical and reproducible training modality, it does not replicate the full complexity of live operative environments—most notably lacking the physiological considerations of anesthesia, hemostasis, and patient variability.

Additionally, the use of a relatively uniform cohort of undergraduate participants—though helpful in minimizing baseline variability—may have influenced perceptions of the model. How the simulator would be perceived by more experienced trainees or clinicians remains unclear. Moreover, the study design was limited by the absence of randomization and blinding.

Future directions to enhance the comprehensiveness of the workshop include incorporating dedicated modules on the psychological and physiological dimensions of amputation care.^36^^,^^37^ Collaborations with patient-centered charities—such as Blesma^38^—may enrich the workshop by embedding lived experiences. Collectively, these strategies could produce doctors who are better equipped to deliver holistic, patient-centered amputation care upon graduation.

## Conclusions

This study contributes to the growing body of evidence supporting the integration of simulation-based teaching into medical education. By equipping medical students with essential skills, confidence, and multidisciplinary knowledge, workshops like this have the potential to improve both training outcomes and patient care standards. As amputation cases continue to rise globally, the adoption of such training models represents a crucial step toward modernizing medical curricula and ensuring that healthcare professionals are well-prepared to meet the evolving needs of patients.

## Role of the funding source

There was no funding source for this study.

## Ethical approval

Ethical approval for this study was granted by the Imperial College Education Ethics Review Process (EERP2425–065). The study was conducted in accordance with the Declaration of Helsinki and UK GDPR regulations. Participation was voluntary with participants providing informed consent for their data to be used towards this research study.

## Appendix Supplementary materials

Image, application 1

Image, application 2

Image, application 3

Image, application 4
