# Supplementary material for: Educational impact of a cost-efficient porcine model for toe amputation simulation training: Enhancing amputation education
Source: JPRAS Open. 2025 Sep 14;46:398–409. doi: 10.1016/j.jpra.2025.09.007 (PMC12604958; doi:10.1016/j.jpra.2025.09.007)
Supplement: Supplementary file 1 [file mmc1.docx]

Appendix A: Pre-Workshop Survey

1. Year of training
   - 1
   - 2
   - 3
   - 4
   - 5 (Final Year)
   - Intercalating
   - FY1
   - FY2
   - Other (What stage of training if you answer other)
2. How would you rate your current theoretical knowledge of the surgical procedure for performing a toe amputation?
   - Very Low
   - Low
   - Neutral
   - High
   - Very High
3. How confident are you in your current surgical skills in performing a toe amputation?
   - Not confident at all
   - Slightly confident
   - Moderately confident
   - Very confident
   - Extremely confident
4. Have you had any prior experience assisting or performing a toe amputation?

- Yes
- No

1. How familiar are you with the surgical instruments used in toe amputation?

- Not familiar at all
- Somewhat familiar
- Moderately familiar
- Very familiar
- Extremely familiar

1. How confident are you in your ability to correctly use the instruments required for a toe amputation?

- Not confident at all
- Slightly confident
- Moderately confident
- Very confident
- Extremely confident

1. How confident are you in managing wound care, stump care, and dressing post-amputation?
   - Not confident at all
   - Slightly confident
   - Moderately confident
   - Very confident
   - Extremely confident
2. How confident are you in identifying key anatomical structures such as joints, tendons, and bones during a toe amputation?

- Not confident at all
- Slightly confident
- Moderately confident
- Very confident
- Extremely confident

1. Please rank the following actions in the order you would perform a toe amputation.

- Locate the Joint by plantarflexing the digit
- Incise the collateral ligaments exposing the joint
- Draw a racket incision leaving a long plantar flap
- Mark the joint
- Cut the long flexor tendon as proximally as possible
- Make a circumferential incision
- Suture the incision closed
- Visualize joint capsule
- Incise the capsule
- Ligate vessels

1. Please select the surgical instruments that are required in a toe amputation.

- Scalpel
- Bone instruments: bone cutter, bone nipper, osteotomes, mallet, and curettes
- Forceps
- Skin hooks
- Scissors
- Retractors
- Needle holders
- Suture material
- Diathermy device
- Indelible pen
- Surgical disinfectant
- Dressing

1. Identify the structure covered by the red box


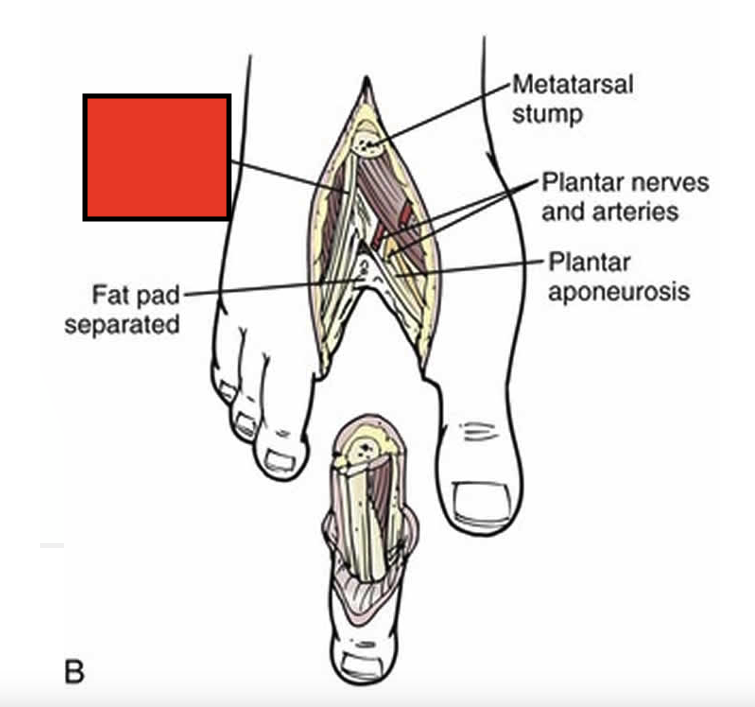


1. Do you consent for your data to be collected for research purposes? All data will be anonymised.

- Yes
- No
