# Supplementary material for: Educational impact of a cost-efficient porcine model for toe amputation simulation training: Enhancing amputation education
Source: JPRAS Open. 2025 Sep 14;46:398–409. doi: 10.1016/j.jpra.2025.09.007 (PMC12604958; doi:10.1016/j.jpra.2025.09.007)
Supplement: Supplementary file 2 [file mmc2.docx]

Appendix B: Post-Workshop Survey

1. How would you rate your current theoretical knowledge of the surgical procedure for performing a toe amputation after this workshop?
   - Very Low
   - Low
   - Neutral
   - High
   - Very High
2. How confident are you in your current surgical skills in performing a toe amputation after this workshop?

- Not confident at all
- Slightly confident
- Moderately confident
- Very confident
- Extremely confident

1. How familiar are you with the surgical instruments used in toe amputation after this workshop?

- Not familiar at all
- Somewhat familiar
- Moderately familiar
- Very familiar
- Extremely familiar

1. How confident are you in your ability to correctly use the instruments required for a toe amputation after this workshop?

- Not confident at all
- Slightly confident
- Moderately confident
- Very confident
- Extremely confident

1. How confident are you in managing wound care, stump care, and dressing post-amputation after this workshop?
   - Not confident at all
   - Slightly confident
   - Moderately confident
   - Very confident
   - Extremely confident

1. How confident are you in identifying key anatomical structures such as joints, tendons, and bones during a toe amputation after this workshop?

- Not confident at all
- Slightly confident
- Moderately confident
- Very confident
- Extremely confident

1. Please rank the following actions in the order you would perform a toe amputation.

- Locate the Joint by plantarflexing the digit
- Incise the collateral ligaments exposing the joint
- Draw a racket incision leaving a long plantar flap
- Mark the joint
- Cut the long flexor tendon as proximally as possible
- Make a circumferential incision
- Suture the incision closed
- Visualize joint capsule
- Incise the capsule
- Ligate vessels

1. Please select the surgical instruments that are required in a toe amputation.

- Scalpel
- Bone instruments: bone cutter, bone nipper, osteotomes, mallet, and curettes
- Forceps
- Skin hooks
- Scissors
- Retractors
- Needle holders
- Suture material
- Diathermy device
- Indelible pen
- Surgical disinfectant
- Dressing

1. Identify the structure covered by the red box


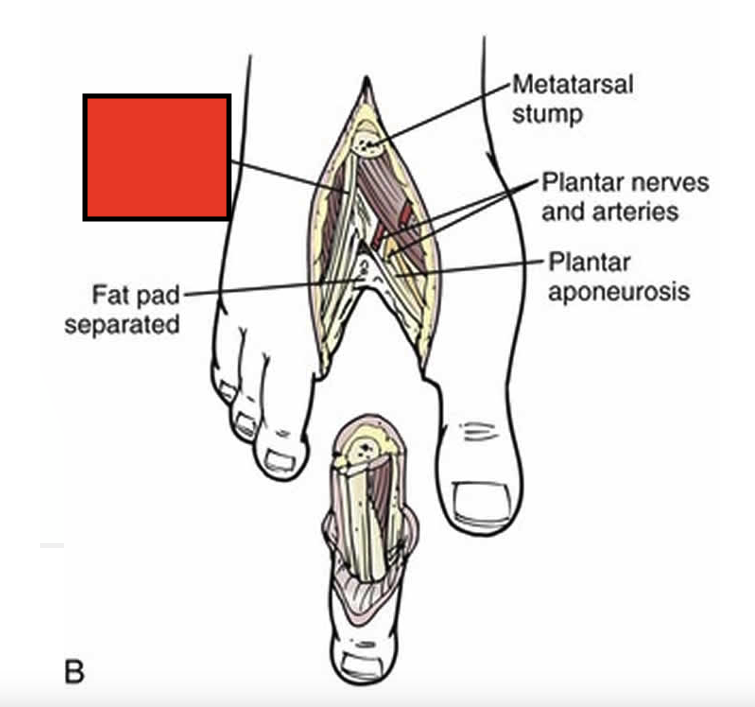


1. My overall comprehension of toe amputation increased due to the training on the model.

- Strongly disagree
- Disagree
- Neutral
- Agree
- Strongly agree

1. What aspects of the training did you find most beneficial or valuable when practising a toe amputation on pork trotters? (Open-ended, optional)
2. Were there any aspects of the training that you found challenging or less effective for a toe amputation on pork trotters? If so, please specify. (Open-ended, optional)
3. Do you feel that the pork trotter toe amputation model adequately facilitates skill acquisition relevant to toe amputation?

- Strongly disagree
- Disagree
- Neutral
- Agree
- Strongly agree

1. How realistic do you feel the material of the pork trotter model is in replicating human tissue?

- Not realistic at all
- Unrealistic
- Neutral
- Realistic
- Highly realistic

1. How would you rate the effectiveness of this workshop in preparing you for assisting/performing a toe amputation in a clinical setting?
   - Not effective at all
   - Slightly effective
   - Moderately effective
   - Very effective
   - Extremely effective
2. The porcine model is useful for learning toe amputation joint incision techniques.

- Strongly disagree
- Disagree
- Neutral
- Agree
- Strongly agree

1. The porcine model is useful for learning toe amputation surgical planning.

- Strongly disagree
- Disagree
- Neutral
- Agree
- Strongly agree

1. The porcine model is useful for learning skin and tissue anatomy of the feet.

- Strongly disagree
- Disagree
- Neutral
- Agree
- Strongly agree

1. The porcine model is useful for learning the steps of toe amputation.

- Strongly disagree
- Disagree
- Neutral
- Agree
- Strongly agree

1. How would you rate your overall experience with using the porcine model for toe amputation training?

- Very poor
- Poor
- Neutral
- Good
- Very good

1. Would you recommend this toe amputation workshop to other medical students/doctors?

- Strongly not recommend
- Not recommend
- Neutral
- Recommend
- Highly recommend

1. Please expand on your answer. (Open-ended, optional)
2. Is there anything else you would like to share about your experience with the toe amputation workshop or other thoughts you may have?
3. Do you consent for your data to be collected for research purposes? All data will be anonymised.

- Yes
- No
