# Supplementary material for: Educational impact of a cost-efficient porcine model for toe amputation simulation training: Enhancing amputation education
Source: JPRAS Open. 2025 Sep 14;46:398–409. doi: 10.1016/j.jpra.2025.09.007 (PMC12604958; doi:10.1016/j.jpra.2025.09.007)
Supplement: Supplementary file 3 [file mmc3.docx]

Appendix C: Global Scoring Assessment Criteria (OSATS)

The following table outlines the global scoring criteria used to assess students in the Toe Amputation station. Scores range from one (lowest) to five (highest), with detailed guidance for each criterion.

| **Criterion** | **Score 1** | **Score 3** | **Score 5** |
| --- | --- | --- | --- |
| **Surgeon Positioning** | Poor positioning and orientation of skill, poor posture, requires instruction | Comfortable position, occasional lapses in orientation or posture, some instruction required | Comfortable position, correct orientation, no instruction required |
| **Instrument Handling** | Awkward handling, unsafe use of sharps, inappropriate movements | Competent and safe use, occasional difficulty with movement or knowledge gaps | Safe and fluid handling, strong knowledge of instruments |
| **Economy of Movement** | Large, unnecessary movements, many readjustments needed | Mostly efficient movements, occasional difficulty | Efficient, appropriate, clear, and precise movements |
| **Tissue Handling** | Inappropriate force, incorrect instruments, evident tissue damage | Mostly careful handling, occasional errors | Completely safe handling, minimal damage |
