# Supplementary material for: Educational impact of a cost-efficient porcine model for toe amputation simulation training: Enhancing amputation education
Source: JPRAS Open. 2025 Sep 14;46:398–409. doi: 10.1016/j.jpra.2025.09.007 (PMC12604958; doi:10.1016/j.jpra.2025.09.007)
Supplement: Supplementary file 4 [file mmc4.docx]

**Appendix D:** Task-Specific Criteria for Toe Amputation

The following table details the task-specific criteria for assessing the Toe Amputation station. Scores range from one (lowest) to five (highest).

| **Criterion** | **Score 1** | **Score 2** | **Score 3** | **Score 4** | **Score 5** |
| --- | --- | --- | --- | --- | --- |
| **Adequate Surgical Planning with Markings** | Unable to perform task | Performs under clear instruction | Minimal instruction, some errors | Independent, minimal errors, minor improvements needed | Independent, no concerns, good efficiency |
| **Incision Placement and Length** | Unable to perform task | Performs under clear instruction | Minimal instruction, some errors | Independent, minimal errors, minor improvements needed | Independent, no concerns, good efficiency |
| **Safe Dissection** | Unable to perform task | Performs under clear instruction | Minimal instruction, some errors | Independent, minimal errors, minor improvements needed | Independent, no concerns, good efficiency |
| **Joint Exposure** | Unable to perform task | Performs under clear instruction | Minimal instruction, some errors | Independent, minimal errors, minor improvements needed | Independent, no concerns, good efficiency |
| **Suture Technique** | Unable to perform task | Performs under clear instruction | Minimal instruction, some errors | Independent, minimal errors, minor improvements needed | Independent, no concerns, good efficiency |
| **Knot Technique** | Unable to perform task | Performs under clear instruction | Minimal instruction, some errors | Independent, minimal errors, minor improvements needed | Independent, no concerns, good efficiency |
| **Motion and Speed** | Unable to perform task | Performs under clear instruction | Minimal instruction, some errors | Independent, minimal errors, minor improvements needed | Independent, no concerns, good efficiency |
| **Overall Outcome (Smooth edges, minimal gapping, aligned flaps)** | Unable to perform task | Performs under clear instruction | Minimal instruction, some errors | Independent, minimal errors, minor improvements needed | Independent, no concerns, good efficiency |
